# Abandonment of surveillance, followed by emergency surgery for a second spontaneous rupture of hepatocellular carcinoma: A case report and review of the literature

**DOI:** 10.1002/ccr3.2097

**Published:** 2019-03-12

**Authors:** Irena Plahuta, Mihael Jelenko, Stojan Potrč, Arpad Ivanecz

**Affiliations:** ^1^ Department of Abdominal and General Surgery University Medical Centre Maribor Maribor Slovenia; ^2^ Faculty of Medicine University of Maribor Maribor Slovenia

**Keywords:** bleeding, emergency liver resection, hepatocellular carcinoma, metabolic syndrome, spontaneous rupture, surveillance

## Abstract

Hepatocellular carcinoma (HCC) develops in the presence of chronic liver disease, and nonalcoholic fatty liver disease is becoming a frequent cause of HCC in developed regions. Spontaneous rupture of HCC (rHCC) is a potentially life‐threatening complication of a tumor. The patient's compliance with surveillance after liver resection is vital for the prevention of rHCC.

## 1 INTRODUCTION

A 50‐year‐old male patient with metabolic syndrome‐related noncirrhotic hepatocellular carcinoma (HCC) suffered from two episodes of spontaneous rupture of HCC (rHCC). After the first rHCC, he abandoned organized surveillance appointments, and the second rHCC occurred from a newly developed HCC. This time, surgical hemostasis and emergency liver resection were used.

Patients with HCC present with a broad spectrum of disease that varies from early stages, often noticed at surveillance, to advanced lesions that can cause symptoms.^1^, ^2^ One of the complications of HCC is the spontaneous rupture of a tumor (rHCC), which has a variable incidence and recently reported rates of 2.3%‐16%.^2^, ^3^, ^4^, ^5^

Rupture of HCC can remain indolent without causing symptoms, but it can also lead to various degrees of abdominal pain and bleeding.^2^ Severe bleeding from rHCC is a potentially life‐threatening condition, and achieving hemostasis is the primary concern.^2^, ^3^ After liver resection, recurrence or de novo formation of HCC often occurs, and the importance of follow‐up and surveillance cannot be stressed enough.^1^, ^6^

This report follows the case of a 50‐year‐old patient who suffered from two episodes of life‐threatening bleeding from two rHCCs within a 6‐year span. He stopped attending organized appointments following radical treatment for the first rHCC and was again admitted to a hospital with bleeding from a newly developed and ruptured HCC.

## 2 CASE PRESENTATION

In 2010, a 50‐year‐old white male was admitted to the Emergency Unit of a tertiary referral center with an acute setting of abdominal pain and with an urge to vomit. His history revealed an obese patient with a body mass index (BMI) of 32 kg/m^2^ and a waist circumference of 120 cm, who was abstinent from alcohol and had arterial hypertension, diabetes mellitus type 2, and dyslipidemia. He was a full‐time employed construction worker and smoked more than 40 cigarettes per day. Later, tests for hepatitis C virus, hepatitis B virus, aflatoxin B1, autoimmune hepatitis, hereditary hemochromatosis, Wilson disease, primary biliary cirrhosis, and alpha‐1 antitrypsin deficiency were all negative.^1^, ^7^ The tumor marker alpha‐fetoprotein (AFP) was elevated at 35.0 IU/mL.^1^ There was no history of prior trauma.

**Figure 1:**
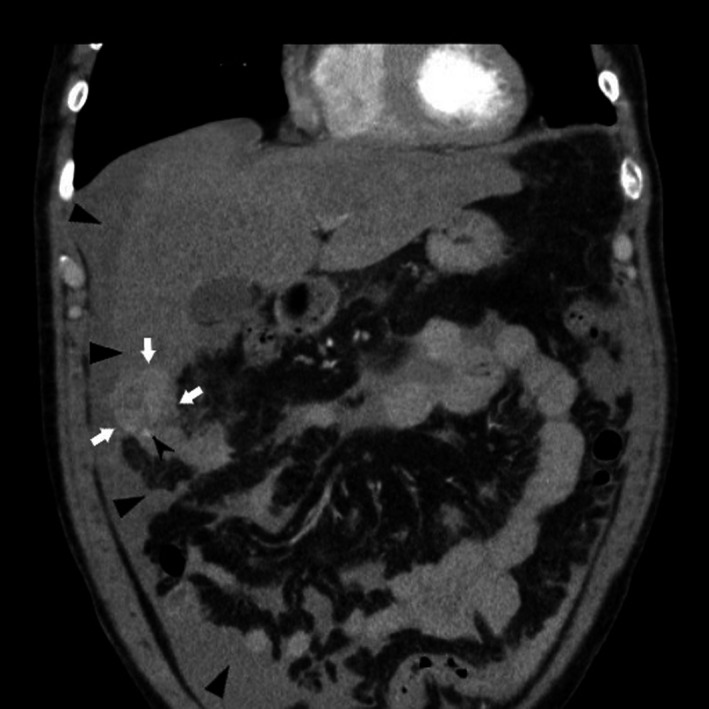
A CT scan in 2010 revealed a ruptured HCC in segment 6 of the liver (white arrows) and a contrast medium extravasation (black arrow). Free fluid (blood) was present in the abdominal cavity (black triangles). CT, computed tomography; HCC, hepatocellular carcinoma

On admission, the patient was pale and normotensive (125/70 mm Hg) and his heart rate was of 90 bpm. A clinical examination showed tenderness on abdominal palpation, which was dominant on the right side. Laboratory results revealed decreased levels of hemoglobin (87 g/L) and hematocrit (0.25). Liver function tests were within the normal range (prothrombin activity of 86%, bilirubin level of 5 μmol/L) or slightly impaired (albumin level of 28 g/L). Ultrasound sonography (US) showed free fluid in the abdominal cavity and a liver tumor in the right hemiliver. A computed tomography (CT) scan later revealed active bleeding from a solitary, vascularized HCC of 4.5 cm in diameter. The tumor was present in segment 6 and protruded from the liver surface (Figure 1). The volume of free fluid in the abdomen was estimated at 1 L.

**Figure 2:**
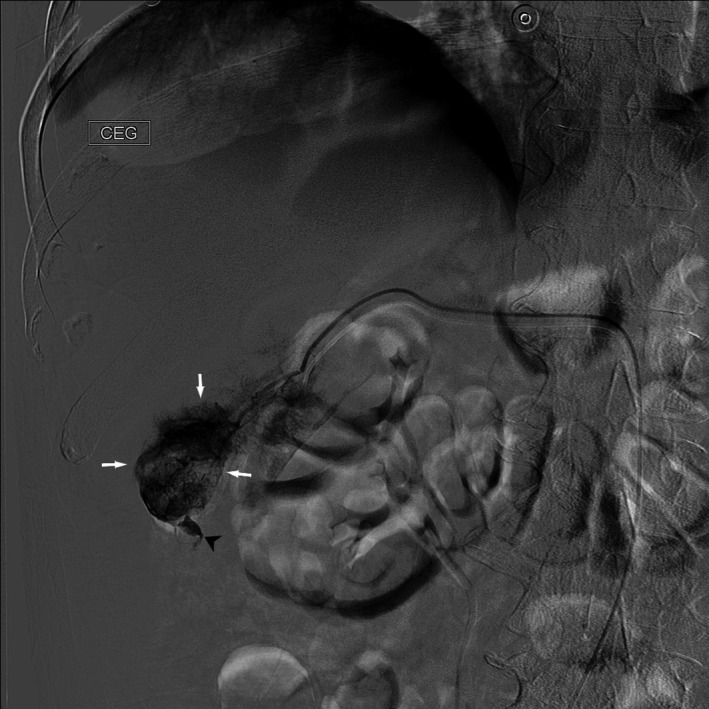
TAE of the bleeding from ruptured HCC. The procedure began with angiography. Contrast medium extravasation could be seen pointing to an active bleeding site (black arrow). A tumor with a pathologically modified vasculature is visible (white arrows). HCC, hepatocellular carcinoma; TAE, trans‐arterial embolization

**Figure 3:**
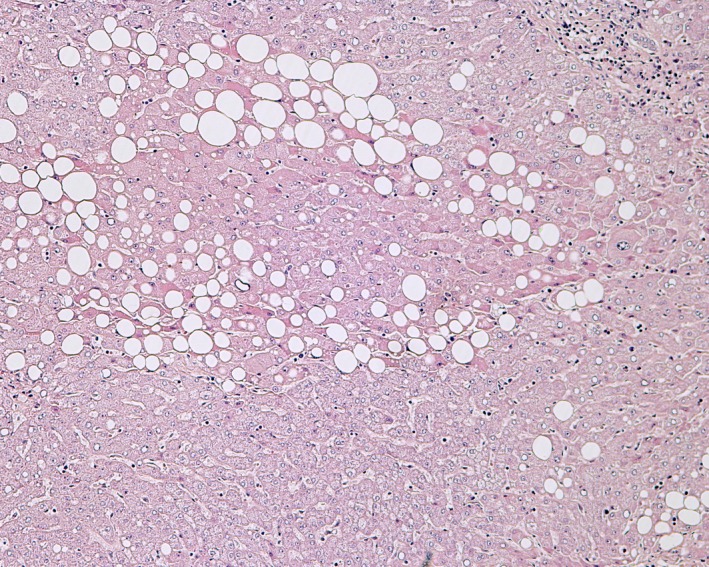
The histopathology of the surrounding nontumorous liver tissue from 2010 revealed nonalcoholic steatohepatitis. Hematoxylin and eosin staining, × 100

The patient's hemodynamic status continued to be stable, and an urgent trans‐arterial embolization (TAE) was performed (Figure 2).^3^ The procedure was successful, and the bleeding stopped. After a brief period of recovery, definitive therapy was considered.^2^, ^3^, ^4^ Four days after TAE, the patient underwent an elective anatomical resection of segment 6. The histopathological examination of the resected specimen confirmed grade 2 HCC with a trabecular growth pattern and no vascular invasion.^8^ A potentially curative R0 resection was achieved with a 23‐mm resection margin. Additionally, the histopathology of the surrounding nontumorous liver tissue (Figure 3) showed no signs of cirrhosis. The nonalcoholic fatty liver disease activity score was 5, revealing nonalcoholic steatohepatitis.^9^ Staging was completed during recovery and showed no signs of systemic dissemination.^1^ The postoperative course was uneventful, and the patient was discharged home on day seven after surgery.

Then, he had regular follow‐ups every three months.^6^ The possibility of liver transplantation in case of a recurrence was proposed to him, although he declined the therapeutic procedure.^10^ Follow‐ups during the first two years revealed no signs of tumor recurrence, and the elevated AFP diminished to a reference value. A change in lifestyle was suggested, including weight loss, special diet, and cessation of smoking.^1^, ^7^ He was prescribed therapy for diabetes, arterial hypertension, and dyslipidemia.^1^, ^7^ However, he did not follow through, and the proposal was unsuccessful. In 2013, for unknown reasons, he abandoned surveillance appointments, with the last visit still detecting no signs of a new HCC alongside laboratory and radiology findings.^1^, ^6^

In 2016, following a 3‐year hiatus from his previous check‐up, the patient was again brought to the Emergency Unit with an acute setting of abdominal pain. He was still obese with a BMI of 31 kg/m^2^ and had all the previously described comorbidities. On examination, he was pale, with a tense, distended abdomen, and a blood pressure of 88/66 mm Hg with a heart rate of 110 bpm. Therefore, aggressive fluid resuscitation was started promptly. Multiple blood tests revealed declining values of hemoglobin (81 g/L) and hematocrit (0.24) with increasing levels of serum creatinine (210 μmol/L) and elevated inflammatory markers, with C‐reactive protein (CRP) levels reaching 113 mg/L. A 58% prothrombin activity, bilirubin level of 27 μmol/L, and albumin level of 24 g/L showed impaired liver function.

**Figure 4:**
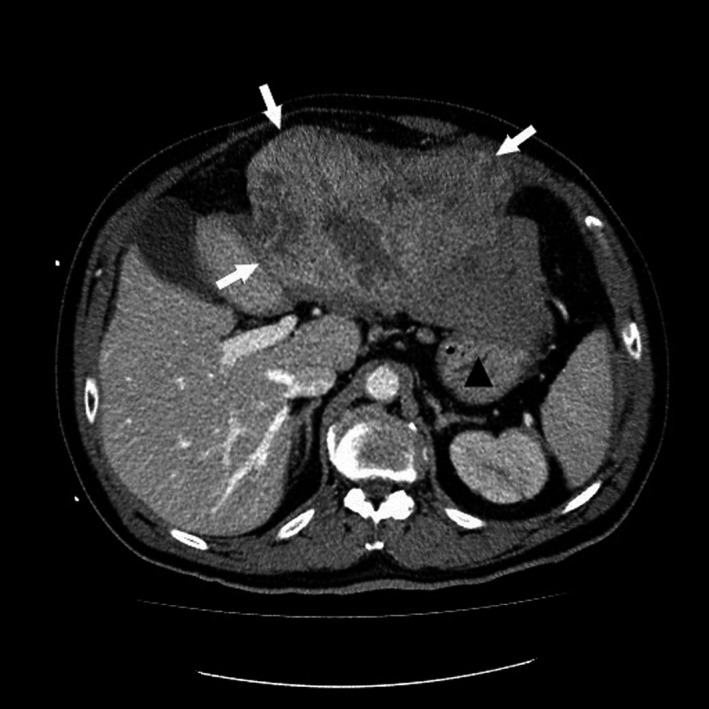
A CT scan in 2016 showed a tumor‐altered left part of the liver (white arrows), with an 8‐cm‐diameter HCC. Note the massive hemoperitoneum (black triangle). CT, computed tomography; HCC, hepatocellular carcinoma

The abdominal US revealed a large mass in the left hemiliver and a collection of free intraabdominal fluid. CT confirmed active bleeding from a rHCC in the left lateral section alongside a massive hemoperitoneum (Figure 4).

To stop the bleeding, an urgent TAE was performed.^3^ However, it failed, and the hemodynamically unstable patient was at once transferred to the operating room (OR). During emergency surgery, a midline laparotomy was performed, revealing an actively bleeding tumor in the left hemiliver and free peritoneal blood estimated at 2.5 L. The arterial branch of the left lateral section of the liver was ligated, and the bleeding stopped.^2^ A hematoma was evacuated from the abdominal cavity followed by closure of the laparotomy incision.

Following successful hemostasis of the ruptured tumor, the patient was transferred to the intensive care unit (ICU). He received multiple units of blood components to correct his anemia and coagulopathy. Soon after, blood tests revealed worsening of acute renal failure (serum creatinine 477 μmol/L). The following day, renal dialysis was needed, and inflammatory markers (CRP 482 mg/L) were highly elevated. The patient was deteriorating despite intensive care support, and the multiple organ dysfunction syndrome (MODS) was worsening. Imaging was repeated, which exposed an expected extensive necrotic region on the side of a tumor. A definitive surgical procedure had been planned, aiming to remove the tumor and the surrounding necrotic tissue. Emergency liver resection was performed two days after surgical hemostasis with an anatomical left lateral sectionectomy.

Histopathology of the tumor confirmed rHCC once again. This time, the tumor was more massive, with a diameter of 8 cm. Multiple tumor thrombi were present in the segmental branches of the portal vein. The trabecular growth pattern was again described, but this time, the histologic grade was defined as 3.^8^ The report of the nontumorous liver parenchyma described a disruption of healthy liver structure, namely, bridging fibrosis.^9^ The staging of the disease was completed during the patient's recovery, and pulmonary metastases were revealed. The resection was R0 locally; however, the systemic spread limited therapeutic options.^1^

After a successful surgical procedure, the patient showed straightforward signs of clinical improvement accompanied by encouraging results from the laboratory findings. He was discharged from the hospital twelve days after liver resection, with a new follow‐up date. According to the Barcelona clinic liver cancer classification, he was classified as stage C (pulmonary metastases at the time of the second rupture) and was therefore eligible for therapy with sorafenib.^1^ A CT scan performed six months later revealed intrahepatic and intraabdominal metastases. However, the patient died from the progression of malignant disease nine months following the second resection and seven years after the potentially curative R0 liver resection of the first rHCC.

## 3 DISCUSSION

Spontaneous rupture with severe bleeding is a potentially life‐threatening complication of HCC.^3^ In this report, we present a patient with not one but two almost identical occurrences of this complication. Reports of such patients are scarce and sparsely described in the literature.^11^ Furthermore, a literature search on rupture of HCC (Tables 1 and 2)^4^, ^5^, ^11^, ^12^, ^13^, ^14^, ^15^, ^16^, ^17^, ^18^, ^19^, ^20^, ^21^, ^22^, ^23^, ^24^, ^25^, ^26^, ^27^, ^28^, ^29^, ^30^, ^31^, ^32^ was undertaken, and the following issues have been found worthy of emphasis.

**Table 1:** Surgical series on ruptured hepatocellular carcinoma, published over the last decade^4^, ^5^, ^12^, ^13^, ^14^, ^15^, ^16^, ^17^, ^18^, ^19^, ^20^, ^21^, ^22^, ^23^, ^24^, ^25^, ^26^, ^27^

Authors Year of publication	Study period	Number of patients with HCC	Number of patients with rHCC (%)	Number of emergency liver resections (%)	Overall early mortality rate of patients with spontaneous rupture of HCC
14 French‐Italian centers					
Schwarz L, et al^12^ 2018	2000‐2012	Not reported	138	24 (18%)	24%
China					
Zhang XF, et al^13^ 2012	2000‐2009	3280	(3.6%)	(13%)	35.6%
Yang H, et al^14^ 2014	2003‐2012	Not reported	132	17 (12.9%)	36.4%
Zhong F, et al^15^ 2016	2004‐2014	Not reported	162	79 (49%)	29%
Zhang W, et al^16^ 2018	2010‐2015	Not reported	137	9 (6.6%)	31.4%
Italy					
Bassi N, et al^17^ 2010	1993‐2008	556	16 (2.9%)	7 (44%)	25%
Tarantino L, et al^18^ 2011	2004‐2010	Not reported	24	2 (8.3%)	Not reported
Japan					
Aoki T, et al^4^ 2014	2000‐2005	49708	1160 (2.3%)	Not reported	Not reported
Sada H, et al^19^ 2016	1986‐2013	1221^a^	64 (5.2%)	1 (1.6%)	Not reported
Tanaka S, et al^20^ 2016	2000‐2013	1980^a^	58 (2.9%)	5 (8.6%)	12%
Malaysia					
Letchumanan VP, et al^21^ 2013	2001‐2010	Not reported	22^a^	14 (63.6%)	13.6%
the Netherlands					
Rijckborst V, et al^22^ 2014	2010‐2014	Not reported	11	1 (9%)	Not reported
South Korea					
Jin YJ^23^ 2013	2003‐2012	1765	(3.5%)	(11.1%)	63.8%
Switzerland					
Joliat GR, et al^24^ 2018	1999‐2015	140^a^	14 (10%)	1 (7%)	0%
Taiwan					
Hsueh KC, et al^25^ 2012	2004‐2010	Not reported	54	19 (35%)	15%
Chan WH, et al^26^ 2016	2010‐2012	2219	117 (5.3%)	15 (13%)	Rupture with shock: 46% Rupture without shock: 13%
Thailand					
Somboon K, et al^5^ 2014	2007‐2012	308	20 (16%)	Not reported	Not reported
UK					
Battula N, et al^27^ 2009	1995‐2005	About 1800	21	5 (24%)	0%

HCC, hepatocellular carcinoma; rHCC, ruptured hepatocellular carcinoma

a Study population of only surgically treated patients.

**Table 2:** Representative surgical case reports of ruptured hepatocellular carcinoma treated with emergency liver resection, published over the last decade^11^, ^28^, ^29^, ^30^, ^31^, ^32^

Authors Year of publication Country	Sex Age (y)	Risk factors for HCC	Presentation	The largest diameter of the ruptured tumor	Management	Outcome	
Veltchev LM^11^ 2009 USA	M 58	Cirrhosis, HBV	Abdominal pain hemodynamical instability	8 cm	Left hepatectomy	3 successive ruptures after 10 y, managed by TAE	
Smith BM, et al^28^ 2009 USA	F 70	Morbid obesity	Abdominal pain	Not reported	Right hepatic partial lobectomy	Discharged after 1 wk	
Rossetto A, et al^29^ 2010 Italy	M 78	HCV and HBV negative	Abdominal pain hemorrhagic shock	4.5 cm	Segmentectomy 6	Local recurrence after 2 mo, treated with TACE. 1 y later without signs of recurrence	
Rombolà F, et al^30^ 2011 Italy	M 73	HCV cirrhosis	Syncope, abdominal pain	12.8 cm	Atypical liver resection	1 mo later in good general condition	
Wszołek J, et al^31^ 2011 Poland	F 66	Obesity	Hemorrhagic shock	9 cm	Right hepatectomy	Discharged on day 17	
Casciaro GE, et al^32^ 2012 Italy	M 87	HCV	Abdominal pain a drop of hemoglobin	10 cm	Left lateral segmentectomy	No signs of recurrence after 3 mo	
Present case 2019 Slovenia	M 50	Metabolic syndrome	2010 abdominal pain hemoperitoneum	4.5 cm	TAE	Elective segmentectomy 6	3 y without signs of recurrence, then lost to surveillance
			2016 abdominal pain hemodynamical instability	8 cm	Ligation of the arterial branch for a left lateral section of the liver	Emergency left lateral sectionectomy	Died 9 mo later due to progression of the disease

HBV, hepatitis B virus; HCC, hepatocellular carcinoma; HCV, hepatitis C virus; TAE, trans‐arterial embolization.

First, the development of HCC is closely related to the presence of chronic liver disease.^1^, ^7^ Nonalcoholic fatty liver disease is becoming an essential cause of HCC in developed regions, and the association with metabolic syndrome (MS) has been described.^1^, ^33^ In most recent Western studies, the reported prevalence of MS‐linked HCC can be up to 20%.^34^, ^35^ Each element of MS increases the HCC risk with an overall risk augmentation of two‐ to threefold.^36^ The presented patient had all the components of MS; he suffered from central obesity, arterial hypertension, diabetes mellitus type 2, and dyslipidemia.^33^ The histopathology of the nontumor parenchyma from the first resection in 2010 (Figure 3) revealed one of the liver manifestations of MS, namely, nonalcoholic steatohepatitis.^9^ Later, the liver parenchyma progressed to bridging fibrosis in 2016.^9^ Moreover, by means of a careful history, excessive alcohol consumption had been excluded.^7^, ^9^ Consequently, a change of lifestyle was recommended to the patient, and he was prescribed drugs for every single MS element.^1^, ^7^ According to the guidelines, he was enrolled in an organized follow‐up protocol, which entailed magnetic resonance imaging or dynamic CT scans every three months during the first two years followed by surveillance every six months.^6^ In addition, it has been a policy of our department that patients who have undergone surgery for cancer have regular, organized check‐ups every three months in the first two years and every six months thereafter. In summary, our patient's lifestyle modification was unsuccessful since the goals of weight loss and smoking cessation were not achieved.^1^, ^7^ Furthermore, the control of arterial hypertension, dyslipidemia, and diabetes mellitus type 2 was also insufficient.^1^, ^7^ Thus, his compliance was not adequate and culminated in the cessation of the surveillance appointments in 2013.^6^

Another issue in this case is the risk factors of spontaneous rupture of HCC. In the literature, several risk factors for HCC rupture have been found, including underlying arterial hypertension, liver cirrhosis, tumor size more than 5 cm, tumor protrusion from the liver surface, vascular thrombosis, and extrahepatic invasion of HCC.^2^, ^37^ In the presented patient, only two of the risk factors had been present at the time of the first rupture, namely, arterial hypertension and protrusion of a tumor from the liver surface. However, at the time of the second rupture, only cirrhosis was absent from the reported risk factors. There was a clear description of other risk factors, namely, arterial hypertension, tumor size more than 5 cm and its protrusion from the liver surface, thrombosis of segmental branches of the portal vein, and pulmonary metastases.^37^ Nonetheless, adequate treatment of every single MS element and attendance at surveillance appointments would have been able to prevent the second rHCC.^1^, ^6^

A point of interest in this case pertains to the challenging treatment of an acute presentation of rHCC. The hemodynamic status of the patient is the main factor in clinical algorithms.^11^, ^13^, ^16^ In unstable patients, resuscitation and hemostasis are the primary concern.^2^, ^3^, ^4^, ^11^, ^13^, ^16^ TAE, surgical hemostasis or emergency liver resection could achieve the latter.^2^, ^3^, ^4^, ^11^, ^13^, ^16^ Following hemodynamic stabilization, staging of HCC and assessment of liver functions could be performed.^2^, ^3^, ^4^, ^11^, ^12^, ^13^, ^16^ Therefore, TAE followed by elective hepatic resection is considered an effective strategy for patients with rHCC.^2^, ^3^, ^4^, ^11^ In 2010, TAE was successfully performed and allowed a period of recovery, followed by a staged, elective hepatectomy.^2^, ^3^, ^4^ In 2016, TAE failed, and the hemodynamically unstable patient was transferred to the OR instantly. Laparotomy with hemostatic maneuvers and packing, hepatic artery ligation, and liver resection have traditionally been considered a reasonable surgical approach for acute hemorrhagic rHCC.^2^, ^3^, ^4^, ^12^ In our patient, only surgical hemostasis (a ligation of an arterial branch for the left lateral section) was possible. He was unfit for liver resection due to hemodynamic instability resulting from significant blood loss, impaired liver function, and MODS.^2^, ^12^ Following surgical intervention and aggressive resuscitation, the bleeding stopped, but MODS was worsening due to the expected liver and tumor necrosis. In these circumstances, emergency liver resection was the best choice. This procedure successfully removed the tumor and necrotic tissue. The benefit of a two‐stage surgery was clear based on the patient's quick recovery without any complications. Nevertheless, recently reported rates of emergency liver resections can be seen in Table 1.^4^, ^5^, ^12^, ^13^, ^14^, ^15^, ^16^, ^17^, ^18^, ^19^, ^20^, ^21^, ^22^, ^23^, ^24^, ^25^, ^26^, ^27^ Representative surgical case reports over the last decade involving emergency liver resection are presented in Table 2.^11^, ^28^, ^29^, ^30^, ^31^, ^32^

Finally, recently reported overall early mortality rates associated with spontaneous rHCC have been from 0% to 63.8% (Table 1).^4^, ^5^, ^12^, ^13^, ^14^, ^15^, ^16^, ^17^, ^18^, ^19^, ^20^, ^21^, ^22^, ^23^, ^24^, ^25^, ^26^, ^27^ Mortality is intricately linked to the presence of liver cirrhosis and severely impaired liver function.^2^, ^12^, ^34^ Our patient successfully underwent emergency surgical procedures since he did not have liver cirrhosis. However, long‐term survival was not achievable due to systemic dissemination of a tumor at the time of the second rupture.^1^

## 4 CONCLUSION

This report revealed the complexity involved in the management of rHCC with severe intraperitoneal hemorrhage. TAE followed by an elective hepatic resection was an effective strategy at the time of the first rHCC. The second manifestation of rHCC, six years after the first, could have been prevented. However, the patient's compliance was not adequate since he did not manage to change his lifestyle, and he did not come to his surveillance appointments. Chronic liver disease progressed, and a new HCC developed in a different location. A two‐stage surgical strategy resulted in a complete recovery of the patient after the second rHCC. However, a systemic spread of the disease precluded long‐term survival at the time of the second rupture.

## CONFLICT OF INTEREST

The authors declare that they have no competing interests. The authors alone are responsible for the content and writing of this article.

## AUTHOR CONTRIBUTION

IP: prepared the review of the literature and wrote the manuscript. MJ: gathered the patient's data. SP: contributed to the review of the manuscript. AI: improved the manuscript and collected graphic material. IP and AI: should be considered joint first authors.

## ETHICS, CONSENT, AND PERMISSIONS

A local Ethics Committee approved this case report.

## References

[1] Forner A , Reig M , Bruix J . Hepatocellular carcinoma. Lancet. 2018;391:1301‐1314. 29307467 10.1016/S0140-6736(18)30010-2

[2] Moris D , Chakedis J , Sun SH , et al. Management, outcomes, and prognostic factors of ruptured hepatocellular carcinoma: a systematic review. J Surg Oncol. 2018;117:341‐353. 29116644 10.1002/jso.24869

[3] Yoshida H , Mamada Y , Taniai N , Uchida E . Spontaneous ruptured hepatocellular carcinoma. Hepatol Res. 2016;46:13‐21. 25631290 10.1111/hepr.12498

[4] Aoki T , Kokudo N , Matsuyama Y , et al. Prognostic impact of spontaneous tumor rupture in patients with hepatocellular carcinoma: an analysis of 1160 cases from a nationwide survey. Ann Surg. 2014;259:532‐542. 23478524 10.1097/SLA.0b013e31828846de

[5] Somboon K , Siramolpiwat S , Vilaichone RK . Epidemiology and survival of hepatocellular carcinoma in the central region of Thailand. Asian Pac J Cancer Prev. 2014;15:3567‐3570. 24870758 10.7314/apjcp.2014.15.8.3567

[6] Verslype C , Rosmorduc O , Rougier P . Hepatocellular carcinoma: ESMO–ESDO clinical practice guidelines for diagnosis, treatment and follow‐up. Ann Oncol. 2012;23:vii41‐vii48. 22997453 10.1093/annonc/mds225

[7] Shiani A , Narayanan S , Pena L , Friedman M . The role of diagnosis and treatment of underlying liver disease for the prognosis of primary liver cancer. Cancer Control. 2017;24:1073274817729240. 28975833 10.1177/1073274817729240 PMC5937237

[8] Schlageter M , Terracciano LM , D’Angelo S , Sorrentino P . Histopathology of hepatocellular carcinoma. World J Gastroenterol. 2014;20:15955‐15964. 25473149 10.3748/wjg.v20.i43.15955 PMC4239483

[9] Brown GT , Kleiner DE . Histopathology of nonalcoholic fatty liver disease and nonalcoholic steatohepatitis. Metabolism. 2016;65:1080‐1086. 26775559 10.1016/j.metabol.2015.11.008 PMC4889547

[10] Sadler EM , Mehta N , Bhat M , et al. Liver transplantation for NASH‐related hepatocellular carcinoma versus non‐NASH etiologies of hepatocellular carcinoma. Transplantation. 2018;102:640‐647. 29319620 10.1097/TP.0000000000002043

[11] Veltchev LM . Spontaneous rupture of hepatocellular carcinoma and hemoperitoneum management and long term survival. J IMAB. 2009;1:53‐57.

[12] Schwarz L , Bubenheim M , Zemour J , et al. Bleeding recurrence and mortality following interventional management of spontaneous HCC Rupture: results of a Multicenter European Study. World J Surg. 2018;42:225‐232. 28799103 10.1007/s00268-017-4163-8

[13] Zhang X‐F , Wei T , Liu X‐M , Lv Yi . Spontaneous tumor rupture and surgical prognosis of patients with hepatocellular carcinoma. Scand J Gastroenterol. 2012;47:968‐974. 22631224 10.3109/00365521.2012.685753

[14] Yang H , Chen K , Wei Y , et al. Treatment of spontaneous ruptured hepatocellular carcinoma: a single‐center study. Pak J Med Sci. 2014;30:472‐476. 24948961 10.12669/pjms.303.4001 PMC4048488

[15] Zhong F , Cheng X‐S , He K , Sun S‐B , Zhou J , Chen H‐M . Treatment outcomes of spontaneous rupture of hepatocellular carcinoma with hemorrhagic shock: a multicenter study. SpringerPlus. 2016;5:1101. 27468402 10.1186/s40064-016-2762-8 PMC4947465

[16] Zhang W , Zhang ZW , Zhang BX , et al. Outcomes and Prognostic Factors of Spontaneously Ruptured Hepatocellular Carcinoma. J Gastrointest Surg. 2018 10.1007/s11605-018-3930-7 30328072

[17] Bassi N , Caratozzolo E , Bonariol L , et al. Management of ruptured hepatocellular carcinoma: implications for therapy. World J Gastroenterol. 2010;16:1221‐1225. 20222165 10.3748/wjg.v16.i10.1221 PMC2839174

[18] Tarantino L , Sordelli I , Calise F , Ripa C , Perrotta M , Sperlongano P . Prognosis of patients with spontaneous rupture of hepatocellular carcinoma in cirrhosis. Updates Surg. 2011;63:25‐30. 21258886 10.1007/s13304-010-0041-8

[19] Sada H , Ohira M , Kobayashi T , Tashiro H , Chayama K , Ohdan H . An analysis of surgical treatment for the spontaneous rupture of hepatocellular carcinoma. Dig Surg. 2016;33:43‐50. 26580332 10.1159/000441531

[20] Tanaka S , Kaibori M , Ueno M , et al. Surgical outcomes for the ruptured hepatocellular carcinoma: multicenter analysis with a case‐controlled study. J Gastrointest Surg. 2016;20:2021‐2034. 27718151 10.1007/s11605-016-3280-2

[21] Letchumanan VP , Lim KF , Mohamad AB . Diagnosis and management of ruptured hepatoma: single center experience over 10 years. Med J Malaysia. 2013;68:405‐409. 24632870

[22] Rijckborst V , Ter Borg MJ , Tjwa ET , et al. Management of ruptured hepatocellular carcinoma in a European tertiary care center. Eur J Gastroenterol Hepatol. 2016;28:963‐966. 27116657 10.1097/MEG.0000000000000652

[23] Jin YJ , Lee JW , Park SW , et al. Survival outcome of patients with spontaneously ruptured hepatocellular carcinoma treated surgically or by transarterial embolization. World J Gastroenterol. 2013;19:4537‐4544. 23901230 10.3748/wjg.v19.i28.4537 PMC3725379

[24] Joliat G‐R , Labgaa I , Uldry E , Demartines N , Halkic N . Recurrence rate and overall survival of operated ruptured hepatocellular carcinomas. Eur J Gastroenterol Hepatol. 2018;30:792‐796. 29538038 10.1097/MEG.0000000000001115

[25] Hsueh K‐C , Fan H‐L , Chen T‐W , et al. Management of spontaneously ruptured hepatocellular carcinoma and hemoperitoneum manifested as acute abdomen in the emergency room. World J Surg. 2012;36:2670‐2676. 22864567 10.1007/s00268-012-1734-6

[26] Chan W‐H , Hung C‐F , Pan K‐T , et al. Impact of spontaneous tumor rupture on prognosis of patients with T4 hepatocellular carcinoma. J Surg Oncol. 2016;113:789‐795. 27062288 10.1002/jso.24245 PMC5071691

[27] Battula N , Madanur M , Priest O , et al. Spontaneous rupture of hepatocellular carcinoma: a Western experience. Am J Surg. 2009;197:164‐167. 18926518 10.1016/j.amjsurg.2007.10.016

[28] Smith BM , Hussain A , Jacobs M , Merrick HW . Ruptured hepatocellular carcinoma in a patient with nonalcoholic steatohepatitis. Surg Obes Relat Dis. 2009;5:510‐512. 19342313 10.1016/j.soard.2008.12.005

[29] Rossetto A , Adani GL , Risaliti A , et al. Combined approach for spontaneous rupture of hepatocellular carcinoma. World J Hepatol. 2010;2:49‐51. 21160956 10.4254/wjh.v2.i1.49 PMC2999262

[30] Rombolà F , Caravetta A , Mollo F , Spinoso A , Peluso L , Guarino R . Sorafenib, risk of bleeding and spontaneous rupture of hepatocellular carcinoma. A clinical case. Acta Medica (Hradec Kralove). 2011;54:177‐179. 22283115 10.14712/18059694.2016.46

[31] Wszołek J , Burenok A . One‐stage emergency right hemihepatectomy due to spontaneous rupture of hepatocellular carcinoma–case report. Pol Przegl Chir. 2011;83:339‐342. 22166551 10.2478/v10035-011-0052-2

[32] Casciaro GE , Spaziani E , Costantino A , et al. Liver resection for hemoperitoneum caused by spontaneous rupture of unrecognized hepatocellular carcinoma. G Chir. 2012;33:221‐224. 22958803

[33] Kaur J . A comprehensive review on metabolic syndrome. Cardiol Res Pract. 2014;2014:943162. 24711954 10.1155/2014/943162 PMC3966331

[34] Viganò L , Conci S , Cescon M , et al. Liver resection for hepatocellular carcinoma in patients with metabolic syndrome: A multicenter matched analysis with HCV‐related HCC. J Hepatol. 2015;63:93‐101. 25646890 10.1016/j.jhep.2015.01.024

[35] Cauchy F , Zalinski S , Dokmak S , et al. Surgical treatment of hepatocellular carcinoma associated with the metabolic syndrome. Br J Surg. 2013;100:113‐121. 23147992 10.1002/bjs.8963

[36] Starley BQ , Calcagno CJ , Harrison SA . Nonalcoholic fatty liver disease and hepatocellular carcinoma: a weighty connection. Hepatology. 2010;51:1820‐1832. 20432259 10.1002/hep.23594

[37] Zhu Q , Li J , Yan JJ , et al. Predictors and clinical outcomes for spontaneous rupture of hepatocellular carcinoma. World J Gastroenterol. 2012;18:7302‐7307. 23326137 10.3748/wjg.v18.i48.7302 PMC3544034

